# Different behaviour of DVL1, DVL2, DVL3 in astrocytoma malignancy grades and their association to TCF1 and LEF1 upregulation

**DOI:** 10.1111/jcmm.13969

**Published:** 2018-11-23

**Authors:** Anja Kafka, Mateja Bačić, Davor Tomas, Kamelija Žarković, Anja Bukovac, Niko Njirić, Goran Mrak, Željka Krsnik, Nives Pećina‐Šlaus

**Affiliations:** ^1^ Laboratory of Neuro‐oncology Croatian Institute for Brain Research School of Medicine University of Zagreb Zagreb Croatia; ^2^ Department of Biology School of Medicine University of Zagreb Zagreb Croatia; ^3^ Research Gate GmbH Berlin Germany; ^4^ Department of Pathology School of Medicine University of Zagreb Zagreb Croatia; ^5^ Department of Pathology University Hospital Center “Sisters of Charity” Zagreb Croatia; ^6^ Division of Pathology University Hospital Center “Zagreb” Zagreb Croatia; ^7^ Department of Neurosurgery University Hospital Center “Zagreb” School of Medicine University of Zagreb Zagreb Croatia; ^8^ Department of Neuroscience Croatian Institute for Brain Research School of Medicine University of Zagreb Zagreb Croatia

**Keywords:** astrocytic brain tumours, Dishevelled, DVL1, DVL2, DVL3, transcription factors TCF1 & LEF1, Wnt signalling

## Abstract

Key regulators of the Wnt signalling, DVL1, DVL2 and DVL3, in astrocytomas of different malignancy grades were investigated. Markers for *DVL1*,*DVL2* and *DVL3* were used to detect microsatellite instability (MSI) and gross deletions (LOH), while immunohistochemistry and immunoreactivity score were used to determine the signal strengths of the three DVL proteins and transcription factors of the pathway, TCF1 and LEF1. Our findings demonstrated that MSI at all three *DVL* loci was constantly found across tumour grades with the highest number in grade II (*P* = 0.008). Collectively, LOHs were more frequent in high‐grade tumours than in low grade ones. LOHs of *DVL3* gene were significantly associated with grade IV tumours (*P* = 0.007). The results on protein expressions indicated that high‐grade tumours expressed less DVL1 protein as compared with low grade ones. A significant negative correlation was established between DVL1 expression and malignancy grades (*P* < 0.001). The expression of DVL2 protein was found similar across grades, while DVL3 expression significantly increased with malignancy grades (*P* < 0.001). The signal strengths of expressed DVL1 and DVL3 were negatively correlated (*P* = 0.002). However, TCF1 and LEF1 were both significantly upregulated and increasing with astrocytoma grades (*P* = 0.001). A positive correlation was established between DVL3 and both TCF1 (*P* = 0.020) and LEF1 (*P* = 0.006) suggesting their joint involvement in malignant progression. Our findings suggest that DVL1 and DVL2 may be involved during early stages of the disease, while DVL3 may have a role in later phases and together with TCF1 and LEF1 promotes the activation of Wnt signalling.

## 1 INTRODUCTION

Astrocytomas are the most common and deadliest form of primary brain tumours.^1^ According to the World Health Organization (WHO) classification, there are four grades of astrocytic brain tumours, considering their histology, molecular characteristics and prognosis.^2^ The least aggressive are pilocytic astrocytomas corresponding to WHO grade I, while diffuse (grade II) and anaplastic astrocytomas (grade III) are malignant types with intrinsic ability to progress to higher grades of malignancy. Of these, glioblastoma multiforme (grade IV) is the most aggressive, fastest‐growing and highly invasive tumour with survival times of about a year.^3^ Despite recent advances in understanding the molecular basis of astrocytoma development and progression, additional research is required to develop more effective therapies.

By its biological characteristics astrocytomas are genetically and pathohistologically a very heterogeneous group of tumours. Complex mechanism of gliomagenesis is the outcome of overlapping between altered signalling pathways. A long‐scale study conducted by The Cancer Genome Atlas (TCGA) revealed numerous data on specific genetic and epigenetic alterations underlying gliomas.^4^ Although it is still not possible to define the exact number and chronology of changes during gliomagenesis, they found that key genes responsible for the formation and progression of astrocytic brain tumours are most frequently involved into deregulated oncogenic pathways: RTK/RAS/PI3K, TP53 and RB.^4^, ^5^ Recent studies have shown that altered signal transduction in Wnt signalling pathway is also involved in the molecular pathogenesis of glial tumours.^6^, ^7^, ^8^, ^9^

The Wnt signalling is an evolutionarily conserved pathway that plays very important roles during embryonic development and tumourigenesis.^10^ In the absence of Wnt ligand, cytoplasmic β‐catenin protein is constantly being degraded by the action of the destruction complex composed of Axin, APC and 2 phosphokinases: casein kinase 1 (CK1) and glycogen synthase kinase 3 (GSK3). Casein kinase 1 and GSK3 phosphorylate β‐catenin, which leads to its proteasomal degradation. Continuous elimination of β‐catenin from the cell prevents β‐catenin from reaching the nucleus, thereby repressing transcription of Wnt target genes.^11^, ^12^

Binding of Wnt ligand to a Frizzled (Fz) receptor and its co‐receptor LRP5/6 activates Wnt/β‐catenin pathway. In this stage the concentration of DVL protein in cytoplasm increases, resulting in recruitment of components of destruction complex to the cell membrane. These events lead to inhibition of β‐catenin phosphorylation, its stabilization and accumulation in the cytoplasm. β‐catenin transfers to the nucleus where it forms complexes with T cell factor 1 (TCF1) and lymphoid‐enhancer factor 1 (LEF1) and in such a fashion activates Wnt target gene transcription.^13^ DVL is the central component of Wnt signalling and key cytoplasmic regulator that rescues β‐catenin from degradation. Three homologous Dishevelled genes (DVL1, DVL2 and DVL3), that show a high degree of similarity, have been found in humans. *Knock‐out* mouse models have shown that each of the DVL proteins can act individually but also in the combination with other family members.^14^ Dishevelled gene proteins function as branching points for the differential interpretation of distinct Wnt ligands.^15^ They are activated by phosphorylation in response to Wnt signals; whereas the ubiquitination of DVL proteins leads to an effective inhibition of the β‐catenin destruction complex.^13^ It has been postulated that DVL overexpression may play a role in the progression of several cancers.^16^, ^17^, ^18^, ^19^, ^20^ As a result it represents a potential target for cancer therapy.

In the present investigation, we searched for changes of all three human *DVL* genes and proteins and tried to define their involvement in specific astrocytoma grade. We were also interested about their effect on Wnt signalling activation and examined transcription factors TCF1 and LEF1.

## 2 METHODS

### 2.1 Tumour specimens

Eighty‐three astrocytoma samples together with corresponding autologous blood tissue were collected with patients’ consents from the Departments of Neurosurgery and Departments of Pathology University Hospital Centers “Zagreb” and “Sisters of Charity”, Croatia and Brain Tumour Tissue Banks from Croatia and Canada. All tumours were studied by certified neuropathologist and classified according to the WHO criteria.^2^ The patients had no family history of brain tumours and did not undergo any cancer treatment (chemotherapy or radiotherapy) prior to surgery. The sample consisted of 17 pilocytic astrocytomas (grade I), 14 diffuse (grade II), 17 anaplastic (grade III) and 35 glioblastomas (grade IV). Thirty‐six patients were female and 47 male. The age of patients varied from 2 to 77 (mean age = 45, median = 44 years). The mean age of diagnosis for females was 49 (median 54.5) and for males 42 years (median 41).

Ethical approval was received from the Ethics Committees School of Medicine University of Zagreb (Case number: 380‐59‐10106‐14‐55/147; Class: 641‐01/14‐02/01) and University Hospital Centers “Sisters of Mercy” (number EP‐7426/14‐9) and “Zagreb” (number 02/21/JG, class: 8.1.‐14/54‐2). Patients gave their informed consent.

### 2.2 DNA extraction

For the genomic DNA extraction, approximately 0.5 g of tumour tissue was homogenized with 1 mL extraction buffer (10 mmol L^−1^ Tris–HCl, pH 8.0; 0.1 M EDTA, pH 8.0; 0.5% sodium dodecyl sulfate) and incubated with proteinase K (100 μg/mL; Sigma‐Aldrich, St. Louis, MO, USA) overnight at 37°C. Phenol chloroform extraction and ethanol precipitation followed. Blood was used to extract DNA from leukocyte. Five millilitre of blood was lysed with 15 mL RCLB (Red Blood Cell Lysis Buffer) (0.16 M NH_4_Cl; 10 mmol L^−1^ KHCO_3_; 10 mmol L^−1^ EDTA; pH 7.6) and centrifuged (15 min/5000 *g*). After overnight incubation with 2 mL SE buffer (Sodium‐EDTA; 75 mmol L^−1^ NaCl; 25 mmol L^−1^ Na_2_EDTA; pH 8), 200 μL 10% SDS (Sodium‐dodecyl sulphate) and 15 μL proteinase K (Sigma, Germany) (20 mg/mL) at 37°C, salting‐out method by isopropanol precipitation followed.

### 2.3 Polymerase chain reaction, multiplex PCR, microsatellite instability, loss of heterozygosity

Genetic changes were tested using PCR amplification of polymorphic microsatellite markers for *DVL1* (D1S468 and D1S243), *DVL2* (D17S960) and *DVL3* (D3S1262) genes to detect microsatellite instability (MSI) and loss of heterozygosity (LOH).^21^ All markers were amplified in a total volume of 25 μL: 5X PCR buffer, 1.5 mmol L^−1^ MgCl_2_, 2.5 mmol L^−1^ of each dNTP, each primer 0.4 μmol, 100 ng DNA, 1 U Taq polymerase (Promega, Fitchburg, WI, USA). PCR conditions and primers for D1S468, D1S243, D17S960 and D3S1262 markers are shown in Table 1.

**Table 1:** PCR conditions for microsatellite markers *D1S468, D1S243, D17S960* and *D3S1262*

Marker	Initial denaturation	Cycles			Final extension	Primers
D1S468	94°C 5 min	94°C 30 s	60°C 30 s	72°C 30 s	72°C 10 min	5′ TTAACCGTTTTGGTCCTACC 3′ 5′ CTCTGACCAGCATTAAAGATTC 3′
D1S243	95°C 5 min	95°C 30 s	56°C 30 s	72°C 30 s	72°C 10 min	5′ CAC ACA GGC TCA CAT GCC 3′ 5′ GCT CCA GCG TCA TGG ACT 3′
D17S960	95°C 5 min	95°C 30 s	58°C 30 s	72°C 30 s	72°C 30 s	5′ CAA TGG AAC CAA ATG TGG TC 3′ 5′ AGT CCG ATA ATG CCA GGA TG 3′
D3S1262	94°C 5 min	94°C 30 s	56°C 35 s	72°C 30 s	72°C 10 min	5′ CGG CCC TAG GAT ATT TTC AA 3′ 5′ CCA GTT TTT ATG GAC GGG GT 3′

Marker D3S1262 was also used for multiplex PCR. It was amplified in the same reaction together with markers for genes SHGC‐68373 (222 bp) 22 and Apex1 (321 bp) 23 that were used as references. Data from the literature indicated that genetic regions where reference genes are located are not changed in glioblastoma and therefore could be used as reference to study oncogenic amplifications.

Genetic alterations were visualized on Spreadex EL 400 Mini gels (Elchrom Scientific, ALLabortechnik, AL‐Diagnostic GmbH, Amstetten, Austria) and stained with SYBR Gold (Invitrogen, Molecular Probes, Eugene, OR, USA). Samples were considered positive for MSI if additional DNA bands appeared in tumour sample, or if the existing DNA bands have changed position in comparison to bands of autologous blood tissue. Absence or significant decrease in the intensity of one polymorphic allele in tumour as compared to the DNA from autologous blood was considered as LOH.

### 2.4 Microsatellite genotyping analysis

Loss of heterozygosity was confirmed by capillary electrophoresis performed on instrument 3730XL (Applied Biosystems Inc.). The 5′‐end of the forward primer was fluorescently labelled with a 6‐FAM dye, and PCR amplification was performed with the AmpliTaq Gold master‐mix (360) (Applied Biosystems Inc.). The labelled PCR products were separated using capillary electrophoresis. Alleles were sized relative to an internal size standard (500 LIZ; Thermo Fisher Scientific, Carlsbad, CA, USA). Raw data and graphical representation of LOH samples were reviewed using GeneMapper 5 and Peak Scanner (Thermo Fisher Scientific).

### 2.5 Immunohistochemistry

In order to establish the levels of expression and cellular localization of DVL1, DVL2, DVL3 gene products immunohistochemistry was used, as well as for testing the upregulation of transcription factors, TCF1 and LEF1. The samples were fixed in formalin, embedded in paraffin, sliced into 4‐μm thick sections, and then fixed onto capillary gap microscope slides (DakoCytomation, Glostrup, Denmark). Briefly, sections were deparaffinized in xylene, rehydrated in decreasing concentrations of ethanol and then microwaved twice for 7 minutes at 400 W in citrate buffer and three times for 5 minutes at 700 W to unmask epitopes. To block endogenous peroxidase activity, cells were fixed in dark with 3% H_2_O_2_ for 10 minutes. Non‐specific binding was blocked by incubating samples with protein block serum‐free ready‐to‐use (Dako, Carpinteria, CA, USA) for 30 minutes at 4°C. Next, the primary antibodies, mouse monoclonal anti‐human for DVL1, TCF1 and LEF1 (1:50; Santa Cruz Biotechnology Inc., Dallas, TX, USA) and rabbit polyclonal anti‐human for DVL2 and DVL3 (1:500; 1:50, Abcam, Cambridge, UK) were applied overnight at 4°C. Slides were then washed three times in phosphate‐buffered saline (PBS), and secondary LINK antibody was applied for 30 min at 4°C. Slides were again washed three times in PBS and incubated with substrate chromogen solution (EnVision™, Dako REAL™) for 30 seconds. Sections were immunostained using streptavidin horseradish peroxidase/DAB (3,3‐diaminobenzidine) (Dako REAL™ EnVision™, Glostrup, Denmark).

The level of expression of DVL1, DVL2 and DVL3 proteins in the healthy brain was determined by using cerebral cortex of human brain (Amsbio, Oxfordshire, UK). We have found that the levels of immunoreactivity of all homologues of the Dishevelled protein family in the healthy brain tissue were very low, and the signal was present only in the cytoplasm.

Negative controls underwent the same staining procedure but without incubating samples with primary antibodies. The frontal cortex of a healthy human brain, placenta, liver tissue and normal bronchial epithelia all served as positive controls. Antibody labelling was analysed by three independent and blinded observers using an Olympus BH‐2 microscope and a digital scanner (NanoZoomer 2.0‑RS; Hamamatsu Photonics, Hamamatsu, Japan). ImageJ software (National Institutes of Health, Bethesda, MD, USA) was used to determine the cell number and the intensity of protein expression. We counted 200 cells in tumour *hot spot* area and performed semi‐quantitative analysis introducing immunoreactivity score (IRS) in order to determine the signal strength. Immunoreactivity score is a factor that best correlates with computational photo analysis and was calculated by multiplying the percentage of cells with a positive signal in the sample (PP score) with staining intensity (SI score). PP score was determined as follows: no immunopositivity in tumour cells = score 0; 1%‐25% positive cells = score 1; >25%‐50% = score 2; >50%‐85% = score 3; >85% = score 4. SI score was assessed in three categories mirroring staining intensities, no staining or weak = score 1, moderate staining = score 2 and strong staining = score 3. Immunoreactivity score in our study ranged from 0 to 12. For statistical analysis needs, IRS values were converted to numbers/symbols: 1 (0/+) (IRS = 0‐4) no expression or very weak expression; 2 (++) (IRS = 6‐8) moderate expression; 3 (+++) (IRS = 9‐12) strong expression.

### 2.6 Statistical analysis

The statistical evaluations for all the obtained data including tumour intracranial localization, pathohistological type and grade, age, sex, genetic findings and protein expression findings were performed with the SPSS v.19.0.1 (SPSS, Chicago, IL, USA) statistical program, with the statistical significance set at *P* < 0.05.

The normality of the distribution of individual parameters within a group was tested using a Kolmogorov‐Smirnov test for normality where low significance (*P* < 0.05) indicates that the distribution significantly differs from normal. Depending on the results of the analysis and the number of patients per group, parametric and nonparametric statistical tests were further used. The differences in the frequency of the chosen parameters were analysed by Pearson's χ^2^ test using Yates's correlation when appropriate. Differences in the values between the four grades were tested by a one‐way variance analysis (ANOVA) in the case of normal distribution. When the assumptions of one‐way ANOVA are not met, Kruskal‐Wallis test was applied. In the case of normal distribution, differences between the two groups were tested by Student's *t* test, and in case of a deviation from normality by the Mann‐Whitney test. When the distribution between individual parameters was normal, we used Pearson correlation, while values derived from Spearman correlation were taken into account if distribution deviated from normality.

### 2.7 Analysis of public databases cBioPortal, COSMIC and GEPIA

In order to test the compatibility of our results we investigated data from publicly available databases. Genetic changes publicly reported on *DVL* genes were assessed from cBioPortal and COSMIC databases,^24^, ^25^ while survival plots were performed from GEPIA database.^26^

## 3 RESULTS

### 3.1 MSI at all three DVL loci was constant across tumour grades while LOHs were associated to higher grades

Our study explored *DVL1, DVL2* and *DVL3* gene alterations in association with tumour grade. The presence of LOH and MSI, two aspects of the genomic instability of tumour cells, was detected in astrocytoma of different malignancy grades.

For *DVL1* gene analysis, two polymorphic microsatellite markers located in the close proximity to *DVL1* gene in chromosomal region 1p36 were selected. Marker D1S468 showed a substantial rate of microsatellite instability (MSI) across all astrocytoma grades, pilocytic (grade I) showed 21.4%, diffuse (grade II) 53.8%, anaplastic (grade III) 36.4% and glioblastoma (grade IV) 17.1%. Contrary LOH was detected only in the highest grade—glioblastomas, in 8.6% of patients (Table 2). Statistical analysis confirmed that the distribution regarding tumour grades was significant. Genetic changes were significantly more distributed to glioblastoma group (*P* = 0.020). Furthermore, our results indicated that MSI was significantly associated to diffuse astrocytomas (grade II) as compared with other astrocytoma grades (*P* = 0.008).

**Table 2:** Polymorphic status of microsatellite markers used and the observed genetic changes of *DVL1*,* DVL2* and *DVL3* genes

Patient no	Grade	DVL1	DVL2	DVL3
		D1S468 & D1S243	D17S960	D3S1262
1	I	HETERO	HOMO	HETERO
2	I	HETERO	HOMO	HETERO
3	I	HETERO	HETERO	HETERO
4	I	HETERO	HETERO	HETERO
5	I	HETERO	HETERO	HOMO
6	I	MSI	LOH	MSI
7	I	HETERO	HOMO	MSI
8	I	MSI	MSI	MSI
9	I	HETERO	HOMO	HETERO
10	I	MSI	HOMO	HETERO
11	I	MSI	HETERO	HETERO
12	I	HETERO	HOMO	HETERO
13	I	HETERO	HETERO	HETERO
14	I	HETERO	HETERO	HETERO
15	II	HETERO	HETERO	HETERO
16	II	HETERO	LOH	HETERO
17	II	MSI^a^	LOH	MSI
18	II	MSI	HETERO	HETERO
19	II	MSI	MSI	HETERO
20	II	MSI^a^	HOMO	HETERO
21	II	HETERO	HOMO	HETERO
22	II	HETERO	HOMO	HETERO
23	II	MSI	HETERO	HETERO
24	II	HETERO	HETERO	HOMO
25	II	MSI	MSI	MSI
26	II	MSI	LOH	HOMO
27	II	MSI	HETERO	HETERO
28	III	HETERO	HOMO	HETERO
29	III	MSI	HETERO	LOH
30	III	LOH	LOH	MSI
31	III	MSI	HOMO	HETERO
32	III	HETERO	HETERO	MSI
33	III	HETERO	HOMO	HETERO
34	III	MSI	HOMO	MSI
35	III	MSI&LOH	HETERO	HETERO
36	III	MSI	HETERO	HETERO
37	III	MSI	LOH	HETERO
38	III	HETERO	HOMO	HETERO
39	IV	HETERO	HOMO	LOH
40	IV	MSI&LOH	HETERO	HETERO
41	IV	HETERO	HETERO	MSI
42	IV	MSI	HOMO	HETERO
43	IV	HETERO	HOMO	HETERO
44	IV	MSI	HOMO	HOMO
45	IV	LOH	HETERO	HETERO
46	IV	MSI	HETERO	LOH
47	IV	HETERO	HOMO	LOH
48	IV	MSI	HETERO	HETERO
49	IV	LOH	LOH	HETERO
50	IV	HETERO	HETERO	HETERO
51	IV	MSI	HETERO	LOH
52	IV	HETERO	HETERO	LOH
53	IV	HETERO	HOMO	MSI
54	IV	HETERO	HETERO	HETERO
55	IV	MSI	MSI	LOH
56	IV	MSI	HOMO	LOH
57	IV	MSI	HOMO	HETERO
58	IV	MSI	HETERO	LOH
59	IV	LOH&MSI	HETERO	HETERO
60	IV	MSI	MSI	LOH
61	IV	HETERO	HETERO	HOMO
62	IV	HETERO	HETERO	MSI
63	IV	LOH&MSI	LOH	HETERO
64	IV	HETERO	LOH	HETERO
65	IV	MSI	HETERO	HETERO
66	IV	HETERO	HETERO	MSI
67	IV	HETERO	HOMO	LOH
68	IV	HETERO	LOH	HETERO
69	IV	MSI	MSI	MSI
70	IV	HETERO	HOMO	HETERO
71	IV	LOH&MSI	LOH	HETERO
72	IV	HETERO	HETERO	HETERO
73	IV	HETERO	HETERO	HOMO

HETERO, heterozygous; HOMO, homozygous; MSI, microsatellite instability; LOH, loss of heterozygosity.

a MSI found by both markers.

The second marker, D1S243, confirmed the constant rates of MSI across all grades: grade I with 7.1%, grade II with 23.1%, grade III with 18.2% and grade IV with 28.6% of MSI, whereas this marker revealed LOH in grades III (18.2%) and IV (9.1%) tumours.

**Figure 1:**
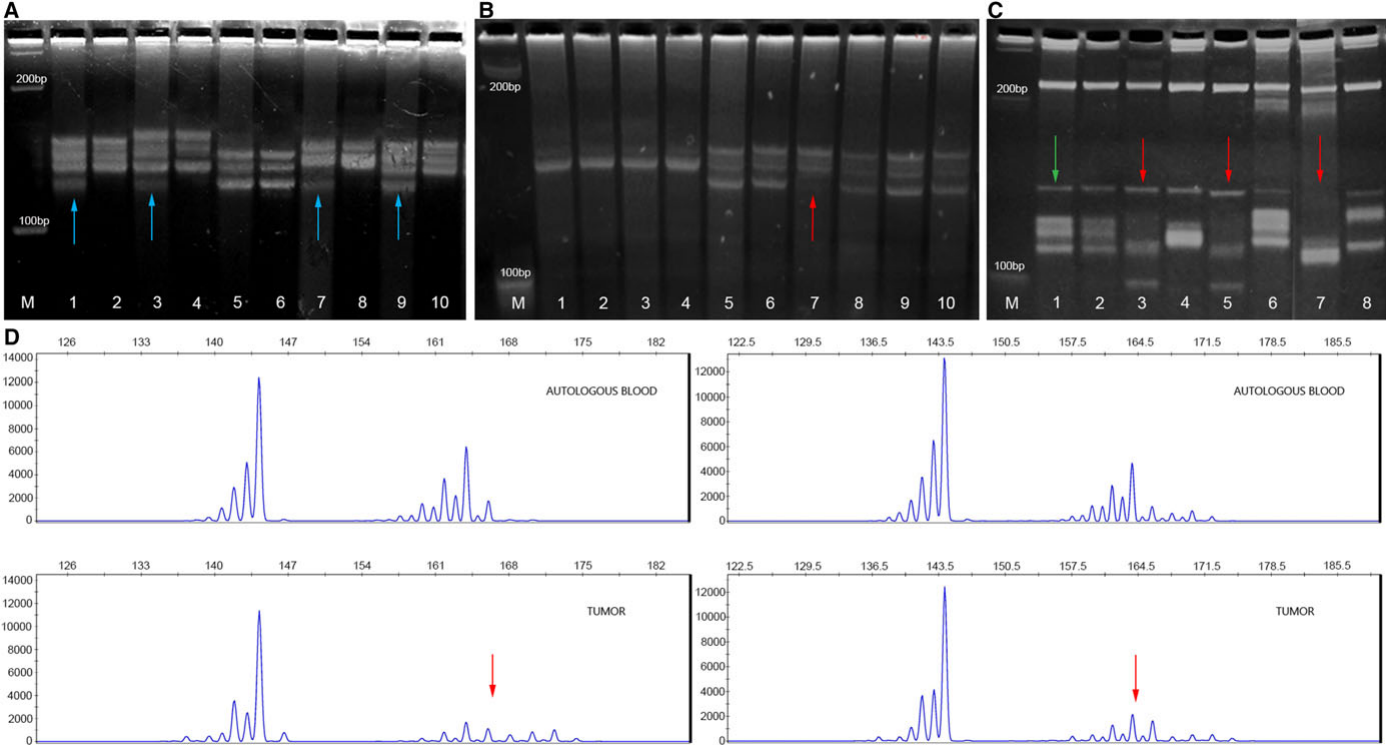
Microsatellite instability (MSI), loss of heterozygosity (LOH) and amplification of A) *DVL1*, B) *DVL2* and C) *DVL3* gene in astrocyoma samples on Spreadex gels. Lane M—100 bp DNA standard; odd lanes show astrocytoma samples; even lanes show corresponding blood samples. Samples demonstrating MSI are indicated with blue arrow; samples demonstrating LOH are indicated with red arrow; samples demonstrating amplification are indicated with green arrow. D) Confirmation of LOH samples by microsatellite genotyping analysis (capillary electrophoresis). Upper figures show DNA samples isolated from the blood, while bottom figures show DNA samples isolated from tumour tissue of the same patient

Collective results for both markers for *DVL1* showed that MSI was detected in 28.6% of pilocytic, 61.5% diffuse, 45.5% anaplastic astrocytomas and in 34.3% of glioblastomas, while LOHs were found in 18.2% of anaplastic and 17.2% of glioblastomas (Figure 1A).

For *DVL2* gene analysis marker D17S960 was selected and it showed a lower rate of MSI in all tumour grades, 7.1% in grade I, 15.4% in grade II, 0% in grade III and 8.6% in grade VI. Again grade II tumours (diffuse) harboured the highest frequency of MSI. However, gross deletions of *DVL2* were detected in all grades (Figure 1B). Thus, grade II and III each harboured 33.3% of LOHs, followed by grade IV with 21.7% and grade I with the lowest frequency of 12.5% (Table 2). The distribution of genetic changes was not significantly associated to any specific grade (*P* = 0.843).

*DVL3* gene was analysed with D3S1262 marker (Figure 1C). Microsatellite instability rate was again relatively constant across different grades (grade I with 21.4%; grade II with 15.4%; grade III with 14.3% and grade IV with 14.3%) while gross deletions were associated only to higher malignancy grades (grade III with 18.2% and grade IV with 31.2%) (Table 2). Statistical analysis revealed that LOH of *DVL3* gene was significantly more frequent in glioblastomas as compared with astrocytomas of lower grades (*P* = 0.007).

To ascertain results on observed genetic changes, genotyping by capillary electrophoresis was additionally performed and LOHs were confirmed as shown in Figure 1D. Taking both approaches together our findings were additionally confirmed.

Besides gross deletions, *DVL3* gene also showed amplifications in 8.6% of glioblastoma patients (Figure 1C). Polymorphic status of used microsatellite markers and observed genetic changes of *DVL1*,* DVL2* and *DVL3* genes are presented in Table 2.

### 3.2 Low rate of somatic mutations and deletions of *DVL* genes is reported on cBioPortal and COSMIC databases

**Figure 2:**
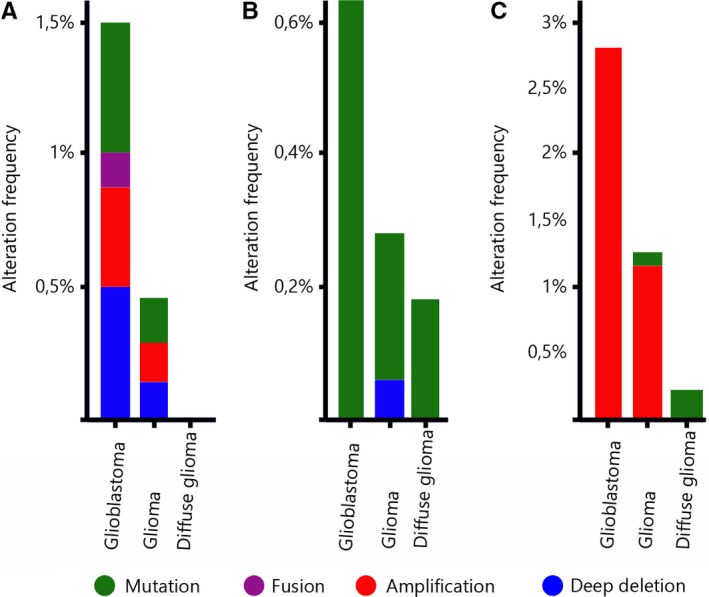
cBioPortal data sets querying 4238 samples in nine studies for each *DVL* gene. Percentages of alteration frequencies of the DVL genes are shown. A) *DVL1*; B) *DVL2*; C) *DVL3*

We tested public datasets downloaded from the Cancer Genome Atlas Database, an open access database that is publicly available at http://www.cbioportal.org and found that the numbers differed to the results of our experiments. Combined collective public datasets from eight studies that are examining low‐grade gliomas and glioblastomas encompassing 4216 samples demonstrate the following distribution: somatic mutations were found in 8/4216 (0.19%) cases for *DVL1*, 12/4216 (0.28%) for *DVL2* and 5/4216 (0.12%) for DVL3. Amplifications are reported mainly for *DVL3* (53 cases showed amplification). Nevertheless, low number of cases with amplified *DVL1* (0.19%) were also reported. Furthermore, deletions of *DVL1* were found in seven glioblastomas and deletions of *DVL2* in two glioblastomas, while deletions of *DVL3* were not reported. A low rate of fusion mutations was also reported. The reported mutations are predominantly missense somatic mutations. It is also obvious that mutational burden is much more frequent in glioblastomas than low‐grade tumours and those amplifications are particularly associated to *DVL3* gene and to glioblastomas. The publicly available data for each *DVL* gene are shown in Figure 2.

### 3.3 High grade tumours expressed less DVL1 protein as compared with low grade ones, the expression of DVL2 protein was similar across grades, while DVL3 expression increased with malignancy grades

**Figure 3:**
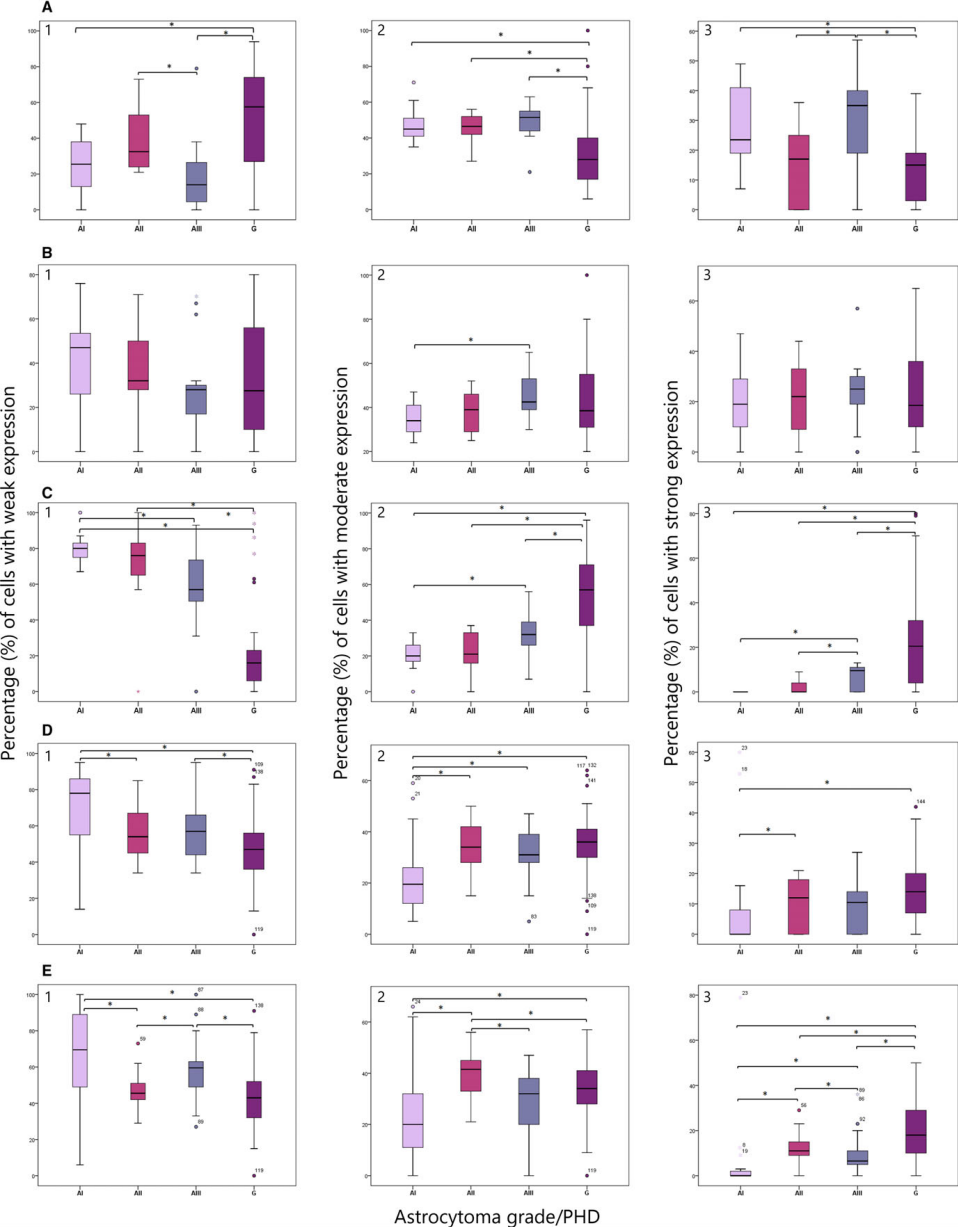
Number of cells with (1) low, (2) moderate and (3) high levels of expression of A) DVL1, B) DVL2, C) DVL3, D) TCF1 and E) LEF1 protein across different malignancy grades. Asterisks (*) denotes statistical significance (*P* < 0.05)

The effect of genetic changes on the protein expressions levels was investigated in the same patients. The expression of DVL1 protein greatly differed among different tumour grades. The highest number of samples with moderate and strong signal was present in the anaplastic astrocytoma group (92.9%) followed by pilocytic (85.7%) and diffuse (70%), while glioblastomas showed low or lack of DVL1 expression in 67.6% of cases. Cell count analysis of DVL1 signal strength showed statistically significant differences in the number of cells with low, moderate and strong expression among different tumour grades (*P* < 0.001). Glioblastomas had significantly more cells with low expression as compared with pilocytic (*P *= 0.001) and anaplastic astrocytomas (*P* = 0.001). Accordingly, glioblastomas had a lower number of cells with strong DVL1 expression as compared with pilocytic (*P* = 0.001) and anaplastic astrocytomas (*P* = 0.001). Moreover, anaplastic astrocytomas showed significantly lower number of cells with low expression levels when compared with diffuse astrocytomas (*P* = 0.008). Negative correlation between DVL1 protein expression and malignancy grades (*r*s = −0.479, *df *= 70, *P* < 0.001) was established (Figure 3A). Of note, in tumours with low levels of DVL1 expression, the signal was predominantly localized in the cytoplasm or in the cytoplasm and cell membrane. In tumours with moderate and strong expression levels of the DVL1 protein, the signal was significantly more frequently localized in cytoplasm and nucleus (*P* < 0.001).

The results of our study demonstrated increased DVL2 expression in all tumour grades when compared with the control tissue. The highest number of samples with moderate and strong signal was present in the group of anaplastic astrocytoma (77.6%) followed by diffuse astrocytoma (70%), glioblastoma (61.7%) and pilocytic astrocytoma (57.2%). As the expression of DVL2 protein was closely the same in all tumour grades, cell count analysis with respect to DVL2 signal strengths did not show significant differences among malignancy grades (Figure 3B). Once again, tumours with the highest proportion of cells with moderate and strong signal had protein located in the cytoplasm and nuclei (*P* < 0.001).

**Figure 4:**
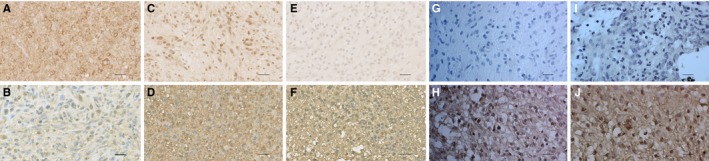
Characteristic immunohistochemical staining of DVL1, DVL2, DVL3, TCF1 and LEF1 proteins in astrocytoma. A) astrocytic brain tumour grade I showing strong cytoplasmic and nuclear staining of DVL1, B) astrocytic brain tumour grade IV showing weak cytoplasmic staining of DVL1, C) astrocytic brain tumour grade I showing strong nuclear staining of DVL2, D) astrocytic brain tumour grade IV showing strong cytoplasmic staining of DVL2, E) astrocytic brain tumour grade I showing lack of expression of DVL3, F) astrocytic brain tumour grade IV showing strong membranous, cytoplasmic and nuclear staining of DVL3, G) astrocytic brain tumour grade I showing weak nuclear expression of TCF1, H) astrocytic brain tumour grade IV showing moderate and strong expression of TCF1, I) astrocytic brain tumour grade I showing weak nuclear expression of LEF1, J) astrocytic brain tumour grade IV showing moderate and strong nuclear expression of LEF1. Scale bar 50 μm

Unlike the two previously analysed proteins, the DVL3 expression increase with malignancy grade was very pronounced. Glioblastoma had highest proportion of cells with moderate and strong expression (82.4%) (Figure 3C), while astrocytomas grade I and II were characterized with weak DVL3 expression, as none of the analysed samples showed more than 50% of positive cells in the tumour *hot spot* area. Anaplastic astrocytomas had 7.2% samples with moderate expression levels. Accordingly, cell count analysis regarding DVL3 signal strength showed significant differences between tumour grades (*P* < 0.001). Glioblastomas had the lowest number of cells showing lack of expression and the highest number of cells showing moderate and strong expression as compared to astrocytoma grades I to III (*P*_1_ < 0.001; *P*_2_ = 0.001; *P*_3_ = 0.013, respectively). Tumours with low signal intensities had predominant cytoplasmic localization, while samples with moderate and strong expression had signal localized in both the cytoplasm and the nucleus (*P* = 0.004) (Figure 3C). This observation suggests that the increased DVL3 expression in glioblastoma leads to more frequent transfer of this protein into the nucleus (Figure 4).

### 3.4 TCF1 and LEF1 protein expression levels increased with astrocytoma grades

Next we asked how the Dishevelleds’ expression is impacting Wnt signalling activation. For this we analysed the expression levels of two transcriptional factors located at the end of the Wnt signalling cascade, TCF1 and LEF1, whose elevated expression indicates the activity of the pathway. Both factors were found to be expressed in our total sample where the levels of both TCF1 and LEF1 expression increased with astrocytoma grades. Accordingly, 61.1% of pilocytic astrocytomas showed the lowest levels of TCF1 expression, while weak or lack of TCF1 expression was confined to 50% of diffuse astrocytomas. The highest expression levels were found in anaplastic and glioblastomas cases, 77.8% and 80% respectively. The signal was present exclusively in the nuclei of tumour cells (Figure 3D).

Statistical analysis for TCF1 protein showed that pilocytic had significantly more cells with low expression levels as compared with diffuse astrocytomas (*P* = 0.040) and glioblastomas (*P* = 0.003). Furthermore, diffuse (*P* = 0.047), anaplastic (*P* = 0.022) astrocytomas and glioblastomas (*P* = 0.001) had significantly higher number of cells with strong TCF1 expression as compared with pilocytic (Figure 3D).

Similar results were obtained for LEF1 protein expression. Low or lack of LEF1 expression was found in 61.1% of pilocytic astrocytomas, while 70% of glioblastomas showed strong LEF1 expression. Almost the same proportions of samples with moderate and strong expression were present in the group of diffuse astrocytoma (88.9%) and glioblastoma (90%), while in anaplastic cases the observed rate was lower (66.6%).

The cell count analysis confirmed the previous observations. Thus, pilocytic astrocytomas had significantly more cells with low LEF1 expression than both diffuse astrocytomas (*P* = 0.006) and glioblastomas (*P* = 0.001) (Figure 3E). The highest number of cells with strong LEF1 expression was in glioblastomas comparing to pilocytic (*P* < 0.001), diffuse (*P* = 0.032) and anaplastic types (*P* = 0.003). Diffuse astrocytomas had significantly more cells with strong LEF1 expression as compared with pilocytic (*P* < 0.001) and anaplastic (*P* = 0.008), while anaplastic had significantly more cells with strong LEF1 expression than pilocytic (*P* = 0.008) (Figure 3E). The results on transcription factors suggest that Wnt activation is accompanying the progression of the disease.

### 3.5 The correlations of molecular findings and clinical parameters

Immunoreactivity score values for DVL1, DVL2 and DVL3 were calculated in order to determine the potential correlations between the expression levels of the proteins.

**Figure 5:**
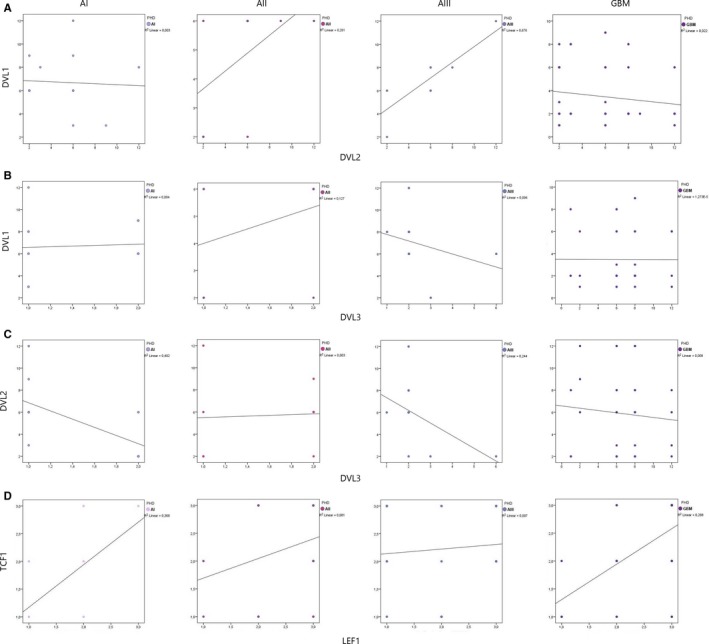
Correlation between A) DVL1 and DVL2, B) DVL1 and DVL3, C) DVL2 and DVL3, D) TCF1 and LEF1 in astrocytic brain tumours grades I‐IV

There was no significant correlation between the expression levels of DVL1 and DVL2 proteins (*P* = 0.799) (Figure 5A). However, DVL1 and DVL3 were significantly negatively correlated in our total sample (*P* = 0.002) (Figure 5B). Lower tumour grades (I and II) showed significantly higher levels of DVL1 expression than high grades (III and IV), while strong DVL3 expression was significantly associated to high‐grade tumours. Comparing the IRS values for DVL2 and DVL3, statistically significant negative correlation was established only for pilocytic astrocytomas (*r* = −0.699, *t* = −3.388, *P* = 0.005), while anaplastic astrocytomas showed a trend of negative correlation for the two proteins (*r* = −0.488, *t* = 0.236, *P* = 0.077) (Figure 5C). Correlation of IRS values for TCF1 and LEF1 revealed significant positive correlation between the two proteins across all tumour grades (*r*_s_ = 0.474, *df *= 92, *P* < 0.001) (Figure 5D).

Bivariate correlation of all analysed proteins revealed a statistically significant positive correlation between DVL3 and TCF1 (*r*_s_ = 0.410, *df *= 30, *P* = 0.020) and DVL3 and LEF1 (*r*_s_ = 0.475, *df *= 30, *P* = 0.006). Correlation of DVL1 and TCF1; DVL1 and LEF1; DVL2 and TCF1; DVL2 and LEF1 showed no statistically significant association between analysed proteins.

Finally we have found that two molecular parameters were significantly associated with aging. Younger patients had a stronger DVL1 expression than older ones (*r*_s_ = −0.226, *df *= 70, *P* = 0.057). On the other hand, older patients had significantly higher DVL3 expression comparing to the younger ones (*r*_s_ = 0.395, *df *= 70, *P* = 0.001). No statistical significance was found in relation to the sex of the patients.

**Figure 6:**
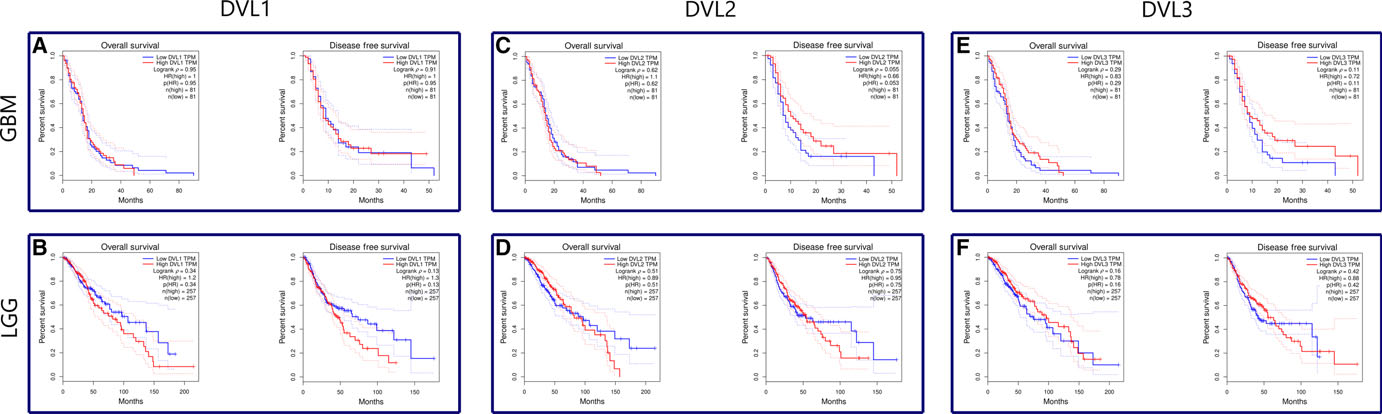
Kaplan‐Meier survival curves generated from GEPIA database for each DVL member. A,B) represent DVL1; C,D) DVL2; E,F) DVL3. A,C,E) demonstrate glioblastoma, B,D,F) low‐grade gliomas

Kaplan‐Meier survival curves were generated from GEPIA database for each DVL member. The curves showed differences among DVL genes expressions and overall and disease‐free survivals. The plots are shown in Figure 6. The plots demonstrated that for low‐grade astrocytomas (Figure 6B,D,F), the low expression levels of DVL1 and DVL2 are correlated to better both overall and disease‐free survival. The same applies for the disease‐free plot of DVL3, while the overall survival plot shows similar survival for both low and high DVL3 levels. Glioblastoma group (Figure 6A,C,E) shows differences. Curves demonstrate similar survival for both high and low DVL1 expression levels, however, disease‐free survival plots for DVL2 and DVL3 show that higher levels of the proteins predict better survival (Figure 6C,E).

## 4 DISCUSSION

Despite advances in molecular and clinical oncology, in the case of astrocytoma, and in particular glioblastoma, no significant progress in the increase of the survival rates has been made over the last decades. Therefore, the identification of novel molecular targets responsible for tumour progression and biomarkers for prediction of disease outcome and response to treatment is extremely requisite. Several novel studies indicate the involvement of Wnt signalling in the molecular pathogenesis of glial tumours.^7^, ^8^, ^9^, ^20^ However, very little is known about the possible association of the proteins involved in the Wnt pathway with tumour malignancy. Especially rare are studies about the roles of DVL1, DVL2 and DVL3 in human astrocytic brain tumours.^27^, ^28^, ^29^ The results of the present study showed that genetic and protein changes of all three Dishevelleds have distinct roles in the process of astrocytoma formation and progression.

We have found the constant rate of MSI across all astrocytoma grades which indicates that genomic instability is part of genetic blueprint of those tumours. Microsatellite instability at *DVL1* locus was detected in 28.6% of pilocytic, 61.5% diffuse, 45.5% anaplastic astrocytomas and 34.3% of glioblastomas. Diffuse astrocytomas displayed significantly higher rate of *DVL1* MSI than other astrocytoma grades (*P* = 0.008). Although at a bit lower frequencies, MSI for *DVL2* was also constantly detected with the exception of anaplastic astrocytomas. Nevertheless, diffuse type again showed the highest number of MSI. For *DVL3* gene MSI was constantly present across different grades.

The lowest frequencies of *DVL1* and *DVL2* MSI were observed for pilocytic astrocytomas which are benign tumours and not prone to malignant progression. However, the highest MSI frequencies were confined to diffuse cases suggesting that the increased genomic instability could be characteristic for astrocytomas that are able to progress to higher grades. It has been known that the increase of mutation accumulation results in an acceleration of the tumour cell evolution.^30^ But besides having effect on cell proliferation, the increased rate of mutations within tumour cells could also lead to cell death. Therefore, only those cells in which the number of mutations does not exceed a certain threshold will have the potential to proliferate. The lower frequencies of MSI found in glioblastomas for all three DVL homologs could be explained by the inability of tumour cells to proceed into much desired apoptosis.^31^

As MSI results from impaired DNA mismatch repair (MMR), several groups^32^, ^33^, ^34^, ^35^, ^36^ analysed MMR genes in astrocytoma of different grades but reported low rate of mutations. Nevertheless, the observed presence of MSI may suggest that yet another mechanism of MMR gene inactivation besides mutations is causative of this molecular phenomenon in astrocytoma. Microsatellites are generally found in non‐coding regions and their role in various malignant diseases is still unexplained.^37^, ^38^ Establishing a pattern of characteristic microsatellite loci in tumours could have predictive potential for tumour behaviour.

The results on gross deletions of the three human *DVL* genes indicate that LOHs of *DVL1* and *DVL3* genes were confined only to the high‐grade malignant group (grades III and IV) while LOHs of *DVL2* were appearing across all grades. Collectively for all DVL genes the number of LOHs was much more frequent in higher‐grade tumours (23 LOHs) than in low grade ones (4 LOHs). Statistical analysis pointed out that deletions of *DVL3* gene were significantly associated to the highest grade (*P* = 0.007) and could be connected to tumour progression.

The chromosomal area on the chromosome 17 where *DVL2* is located was often deleted in our study. This is consistent with several earlier studies investigating aberrations of chromosome 17 in various tumour types including lung, breast, ovarian and brain tumours.^39^, ^40^, ^41^, ^42^, ^43^ Lee et al^39^ found LOH on locus 17p in 50% of anaplastic astrocytomas and glioblastomas, but did not detect it in tumours of lower grades. We too, have found LOH to be more frequent in malignant types (grades III and IV) or those types prone to malignancy (grade II).

Genetic changes of *DVL3* gene suggest its involvement in the process of glioma progression. We noticed that the percentage of LOH increased with the malignancy grade (grade III with 18.2% and grade IV with 31.2%), while the presence of MSI was slightly decreasing in higher grades (grade I with 21.4%; grade II with 15.4%; grade III with 14.3% and grade IV with 14.3%). Statistical analysis revealed that LOH of *DVL3* was significantly more frequent in glioblastomas than in lower‐grade tumours (*P* = 0.007). We also found the amplification of *DVL3* gene in 8.6% of glioblastoma which could indicate different cellular roles for this gene. Other research groups showed similar results. Hu et al^44^ detected 30% of glioblastomas with LOH in the chromosomal region 3q where *DVL3* is located. On the other hand, Hui et al^45^ investigated amplifications in the glioblastoma genome using the aCGH, and found that the region 3q was amplified in 14.3% of the tumour.

Data from cBioPortal and COSMIC databases^24^, ^25^ report on low rate of somatic mutations, fusions and deep deletions of all *DVL* genes. However, the mutations that have been found are much more frequent in glioblastomas than low‐grade tumours. The reported rate of amplifications of *DVL3* is similar to the ones we found.

Taken together, we showed that MSI occurred constantly across all four tumour grades, with more frequent incidence in lower astrocytoma grades, suggesting its association with tumour formation. Loss of heterozygosity was found to be often present in anaplastic astrocytoma and glioblastoma and therefore could be involved in the process of tumour progression as a background mechanism for inactivation of tumour suppressor genes. All things considered, it is clear that LOH and MSI contribute to the genomic profile of astrocytoma.^46^, ^47^, ^48^, ^49^The levels of DVL1, DVL2 and DVL3 protein expressions were studied as the functional consequences of each Dishevelled homologue on astrocytoma tumour formation are still not fully clarified, and the data from the literature are contradictory. Most studies report on gene amplifications and high expression of DVL proteins in general,^17^, ^50^ but others describe large deletions of these genes as well.^51^, ^52^ Although our results generally showed higher DVL1 expression levels when compared to the control tissue, when dividing the sample to malignancy grade groups it became obvious that the expression levels of DVL1 differed significantly across tumour grades (*P* < 0.001). Glioblastomas displayed predominant low DVL1 expression, while anaplastic, pilocytic and diffuse astrocytomas showed predominantly moderate and strong signals. Our results indicate that expression levels of DVL1 dropped in the highest grade. Glioblastomas had significantly more cells with low expression and significantly less cells with strong expression as compared with pilocytic (*P* < 0.001) and anaplastic astrocytomas (*P* < 0.001). Moreover, diffuse astrocytomas had significantly more cells with low expression when compared to anaplastic cases (*P* = 0.008). Negative correlation between DVL1 protein expression and malignancy grades (*r*_s_ = −0.479, *df *= 70, *P* < 0.001) was established indicating that elevated protein expression could be attributed to early events. Also younger patients had a stronger DVL1 expression than older ones (*r*_s_ = −0.226, *df *= 70, *P* = 0.057). We also noticed that in tumours with low DVL1 expression, the signal was predominantly localized in the cytoplasm or in the cytoplasm and cell membrane, while in those with moderate and strong expression, the signal was confined to cytoplasm and nucleus (*P* < 0.001). Several authors showed that *DVL1* gene amplification and elevated protein expression are involved in the development of breast and prostate cancer^16^, ^53^, ^54^ accompanied with positive correlation between DVL1 and β‐catenin location in the nucleus.^53^

DVL2 was also upregulated in all tumour grades in comparison to control samples. The highest number of samples with moderate and strong signal was present in anaplastic astrocytoma (77.6%) followed by diffuse astrocytoma (70%), glioblastoma (61.7%) and pilocytic astrocytoma (57.2%). As the expression of DVL2 protein was quite similar across tumour grades, cell count analysis of signal strengths did not show significant differences among malignancy grades. Once again, tumours with the highest proportion of cells with moderate and strong signal had protein located in the cytoplasm and nuclei (*P *< 0.001). A study by Pulvirenti et al^55^ report on increased DVL2 expression in 70% of glioblastomas. The authors confirmed the role of DVL2 in human glioblastoma by *DVL2* gene attenuation using RNAi. Furthermore, when DVL2 activity was decreased in the glioblastoma cells it prevented tumour development in the immunodeficiency mice. There are also studies showing that DVL2 is involved in the progression of colon cancer.^56^

The clear picture on the increase of the expression levels with the malignancy grade was obtained for DVL3. In all grade I and II astrocytomas samples, DVL3 expression was characterized as weak, while 82.4% of glioblastomas showed moderate and strong expression levels. Accordingly, cell count analysis regarding DVL3 signal strength showed significant differences among tumour grades (*P* < 0.001). Glioblastomas had the lowest number of cells showing lack of expression and the highest number of cells showing moderate and strong expression as compared to other grades (*P*_1_ < 0.001; *P*_2_ = 0.001; *P*_3_ = 0.013 respectively). Unlike the two previously analysed proteins, the DVL3 expression increase with malignancy grade was very pronounced. Of note is that older patients had significantly higher DVL3 expression comparing to the younger ones (*r*_s_ = 0.395, *df *= 70, *P* = 0.001). There was no significant correlation between the expression levels of DVL1 and DVL2 proteins (*P* = 0.799). However, DVL1 and DVL3 were significantly negatively correlated in our total sample (*P *= 0.002). Stronger DVL1 expression was associated to low‐grade tumours and strong DVL3 expression to high‐grade tumours. As the correlation between the decreased protein expression of DVL3 and the presence of gross deletions has not been established, there is a possibility that the observed LOHs are the reflection of tumour heterogeneity occurring only in certain glioblastoma subtypes.

With regard to subcellular localization of DVL3 we noticed that tumours with low signal intensities showed predominant cytoplasmic localization, while moderate and strong expression was usually localized in both the cytoplasm and the nucleus (*P* = 0.004). This observation suggests that the increased DVL3 expression in glioblastoma leads to more frequent transfer of this protein into the nucleus. The data from the literature report on two cellular pools of DVL: one in the cytoplasm and another in the nucleus. Gan et al^57^ found that DVL3 was accumulated in the nucleus in 36% of the analysed colon cancer samples. It seems that DVL migrates from one cell compartment to another after its concentration reaches a level necessary for signal transduction. As our results of the bivariate correlation between DVL3 and transcription factors TCF1 and LEF1 showed that the proteins were significantly positively correlated, we can assume that the increased DVL3 expression could ultimately lead to enhanced transcriptional activity of target genes.

Similar to us, two studies^17^, ^27^ also showed different behaviour of DVL members in human tumours. Sareddy et al^27^ in astrocytoma showed that the expression of DVL3 mRNA was elevated in all three malignancy grades, while the expression of DVL1 and DVL2 mRNAs was not detected. Authors also noticed a strong positive correlation between DVL3, β‐catenin and LEF1 at the mRNA level, indicating increased activity of the Wnt signalling pathway. Uematsu et al^17^ observed increased DVL3 expression in non‐small cell lung cancer, while expression of DVL1 and DVL2 was not detected. Other papers studied Dishevelled expression^29^, ^58^ and found that it increased with the histopathological grade. Dishevelled was positively correlated with the proliferation index Ki‐67, tumour invasion index MMP‐9 and β‐catenin, and transferred to the nucleus which indicated its involvement in the proliferation and invasion processes. Our group previously analysed DVL1 and DVL3 proteins in metastases from lung to the brain. The results showed elevated DVL1 (87.1%) and DVL3 (90.3%) expression and cytoplasmic and nuclear localization of the signal.^59^ It is important to discuss the potential roles that each DVL member plays in signalling circumstances. Such studies are rare and how DVLs conduct activity still remains poorly understood. Firstly, it has been proposed that because of the high similarity of DVL genes in mice and humans, members play equal roles and there may be redundancy in how they work. However, Sharma et al,^60^ based on mouse knockout models, indicated that each DVL member may have a unique role to play. To elucidate the specific roles of DVL genes, rescue studies on double knockout mice were conducted. Double knockout of DVL1 and DVL3 did not display neural tube defects which indicate that each DVL has a unique role to play. Another point in favour of different roles is the aberrant expression of DVL genes linked to different human disorders. DVL1 has been reported to be a candidate gene for human development disorder Schwartz‐Jampel syndrome, Charcot‐Marie‐Tooth disease type 2A and DiGeorge Syndrome, while DVL3 have been linked to Hirschsprung's disease.

Interestingly, Sharma et al^60^ performed a TCGA analysis of DVL1, DVL2 and DVL3 expression across of four different types of cancer including glioblastoma and found that DVL RNA expression in majority of those cancers did not differ as compared to adjacent normal tissues except for DVL1. The authors report on downregulation of DVL1 RNA in glioblastoma and upregulation of DVL3 RNA in lung cancer. This is very similar to our results and may suggest the importance of the post‐transcriptional regulation of DVL proteins. The activation of Wnt signalling is primarily mediated by transcription factors from the TCF/LEF family. It is well known that the TCF1 and LEF1 proteins can have truly diverse effects in mediating signalling. Their impact on activation or repression of the target genes depends on the protein partner they interact with. Numerous studies investigated TCF1 and LEF1 transcription factors in various tumour types including colon, kidney, breast, adenocarcinoma, melanoma, several different types of leukaemia and lymphoma^60^, ^61^, ^62^ and concluded that their expression is important in maintaining the physiological functions of cells, while their overexpression causes invasion of tumour cells^60^ and promotes epithelial‐to‐mesenchymal transition.^63^

The results of our study showed that both TCF1 and LEF1 showed elevated expression and this upregulation indicates that the Wnt pathway was activated. Moreover, the levels of both TCF1 and LEF1 expression increased with astrocytoma grades. Astrocytomas of higher grades had significantly higher number of cells with strong TCF1 expression as compared with lower grades (*P* = 0.022 anaplastic and *P* = 0.001 glioblastoma). Similarly, the highest number of cells with strong LEF1 expression was confined to glioblastomas as compared with pilocytic (*P* < 0.001), diffuse (*P* = 0.032) and anaplastic types (*P* = 0.003). Correlation of IRS values for TCF1 and LEF1 revealed significant positive correlation between the two proteins across all tumour grades (*r*_s_ = 0.474, *df *= 92, *P* < 0.001).

Positive correlation was established between DVL3 and both TCF1 (*r*_s_ = 0.410, *df *= 30, *P* = 0.020) and LEF1 (*r*_s_ = 0.475, *df *= 30, *P* = 0.006) indicating once again DVL3 link to progression.

There are several studies investigating the role of Wnt signalling in human astrocytoma.^27^, ^64^ Sareddy et al^65^ demonstrated strong expression of TCF4 and LEF1 in diffuse, anaplastic astrocytomas and glioblastomas. They showed that the activation of the transcription factors was induced by β‐catenin migration into the nucleus. A positive correlation was also found between the expression level of TCF4 and LEF1 compared to the astrocytoma grade. Although we analysed another member of TCF family, the findings are still consistent to our results.

Our previous study^66^ on beta‐catenin expression in neuroepithelial brain tumours of different grades strengthens the claim on Wnt signalling pathway's activity. We have shown that beta‐catenin was upregulated in 53.1% of samples. Moreover, in 21.4% of glioblastomas beta‐catenin was accumulated exclusively in the nucleus, while in 38.1% in both the cytoplasm and nucleus.

## 5 CONCLUSIONS

Together the results of this study show that important regulatory components of the Wnt signalling pathway, DVLs, have different roles in astrocytomas. Our study revealed that LOH and MSI contribute to the genomic profile of astrocytoma. Furthermore, a noticeable difference in the expression levels of DVL proteins in different tumour grades was established. We have found that DVL1 and DVL2 in astrocytic brain tumours act in one fashion and may be involved during early stages of the disease, while DVL3 behaves differently being involved in the progression while having activating effect on TCF1 and LEF1 transcription factors of Wnt pathway.

## ETHICS APPROVAL AND CONSENT TO PARTICIPATE

This study was conducted in compliance with the declaration of Helsinki regarding ethical principles for medical research involving human volunteers. This research was approved by Ethics Committees of the School of Medicine University of Zagreb and University Hospital Centers “Sisters of Charity” and “Zagreb”.

## ACKNOWLEDGEMENT

We thank Brain Tumour Foundation of Canada for donating brain tumour samples.

## AUTHOR CONTRIBUTION

AK contributed to data acquisition and analysis, performed experimental work, wrote the manuscript and revised it for important intellectual content. MB performed experimental work, participated in data collection, interpretation and analysis. DT participated in tumour sample analysis and results interpretation and revised the manuscript for important intellectual content; KŽ participated in tumour sample analysis and revised the manuscript for important intellectual content; AB contributed to the data interpretation, manuscript editing, revised the manuscript for important intellectual content. NN contributed to data acquisition, participated in tumour sample analysis and manuscript editing. GM participated in tumour sample analysis and revised the manuscript for important intellectual content; ŽK contributed to data acquisition, the interpretation of the results and manuscript editing. NPŠ produced the idea, designed the study, contributed to analysis and interpretation of the results, wrote the manuscript and revised it for important intellectual content, and approved the final version of the manuscript. All authors read and approved the final manuscript.

## CONFLICT OF INTEREST

All authors declare that they have no conflicts of interest.
